# Functional outcome of non-surgical and surgical management for de novo degenerative lumbar scoliosis: a mean follow-up of 10 years

**DOI:** 10.1186/s13013-017-0143-x

**Published:** 2017-12-05

**Authors:** Sayf S.A. Faraj, Tsjitske M. Haanstra, Hugo Martijn, Marinus de Kleuver, Barend J. van Royen

**Affiliations:** 1 Department of Orthopaedic Surgery, VU University Medical Center, De Boelelaan 1117, 1081HV Amsterdam, The Netherlands; 2 Department of Orthopedics, Radboud University Medical Center, Huispost 611, 6500HB Nijmegen, The Netherlands

**Keywords:** De novo degenerative lumbar scoliosis, Adult scoliosis, Adult spinal deformity, Treatment, Surgical, Non-surgical, Functional outcome

## Abstract

**Background:**

No studies have evaluated the long-term results of non-surgical and surgical management in de novo degenerative lumbar scoliosis (DNDLS). This study reports on the long-term functional outcome of patients being treated for DNDLS by non-surgical and surgical management.

**Methods:**

This is a retrospective review of a single center database of DNDLS patients that underwent surgical or usual non-surgical management between 1996 and 2007. In a total of 88 patients, 50 (57%) underwent non-surgical and 38 (43%) surgical management. Baseline demographic, radiological-, clinical-, and surgical-related variables were collected. An Oswestry Disability Index (ODI) 2.0 questionnaire was sent to all patients after written informed consent.

**Results:**

Twenty-nine of 88 patients participated in the study, 15 (52%) had undergone surgical and 14 (48%) non-surgical management with a mean follow-up of 10.9 years (range 8–15 years). There were no significant differences (*p* > 0.05) between non-surgical and surgical patients at baseline for age, body mass index, coronal Cobb angle, and clinical data. None of the non-surgical patients had undergone surgery during follow-up. In the surgical group, 40% had revision surgery. There was no significant difference in ODI total scores between groups at final follow-up (*p* = 0.649). A larger proportion of patients in the non-surgical group reported an ODI total score of ≤ 22, reflecting minimal disability (43 versus 20%; *p* = 0.245).

**Conclusions:**

This is the first study that describes the long-term 10-year functional outcome of non-surgical and surgical management in a cohort of patients with DNDLS. No significant difference in functional outcome was found between groups after a mean follow-up of 10 years. Despite the significant potential for selection bias, these results indicate that non-surgical management of patients with DNDLS may lead to adequate functional outcome after long periods of time, with no crossover to surgery. Further study is warranted to define which patients may benefit most from which management regimen.

## Background

De novo degenerative lumbar scoliosis (DNDLS) is a condition in which a lumbar scoliotic curve develops after the fifth decade as a result of degenerative changes of the spine [1]. DNDLS is generally moderately symptomatic, but it can result in severe back pain and neurological symptoms including leg weakness and numbness, thereby resulting into severe functional impairments [2–4]. The prevalence of DNDLS in the low back pain population has been reported to be 15% [5], and 68% in asymptomatic adults over the age of 60 years [6]. In light of the aging population, the prevalence of DNDLS will continue to increase [7, 8]. For this reason, the identification of proper ways of management has gained in urgency.

Non-surgical management of DNDLS with exercise therapy, the use of non-steroidal anti-inflammatory drugs, steroid injections, and narcotics has shown to be inadequate in providing relief of symptoms [9, 10]. As a result, a large subset of patients ultimately reach the point of undergoing lumbar spinal surgery, a decision that depends on progression of the deformity, neurological symptoms, functional limitations, and patient and surgeon preference [11, 12]. Despite the fact that these factors are patient-specific, surgical management is a generally accepted and a commonly performed intervention for this disorder. However, surgical treatment for DNDLS is known to be associated with high complication and revision rates [13].

Several studies have compared surgical with non-surgical management for DNDLS. Surgery has repeatedly been shown to be superior in relieving pain and improving function over a follow-up of 2 years [14–16]. However, emphasis on healthy and vital aging, in which elderly want to maintain their functional abilities, reflects the need to evaluate both management regimens after long periods of time. Even though surgery is widely used, to the best of our knowledge, no studies are available in the peer-reviewed literature that have evaluated both strategies over a long-term follow-up. The objective of this study is, therefore, to evaluate long-term functional outcome of surgical and non-surgical management in patients with DNDLS, exceeding a mean follow-up of 10 years. The focus will be patient centered, hence, using a validated patient-reported outcome measure to evaluate function.

## Methods

### Study design and patient population

This is a single-center observational cohort study of patients who presented between 1996 and 2007 with symptomatic DNDLS. Inclusion criteria were age ≥ 50 years and a Cobb angle of 10°–55° in the coronal plain with an apex of the main curvature located in the lumbar region (L1-L5). Patients with a history of juvenile, adolescent idiopathic scoliosis, neuromuscular spinal abnormalities, or metabolic spinal pathology were excluded. A total of 88 patients met the inclusion criteria. Baseline demographic and radiographic findings of this study population have been reported previously [17].

In this group of 88 patients, 50 (57%) underwent non-surgical and 38 (43%) underwent surgical management based upon a consent process between patient and surgeon and was ultimately guided by patient choice and surgeon preference. Patients being treated surgically underwent decompression, short fusion (≤ 3 levels), or long fusion (≥ 4 levels) with pedicle screw fixation. Standardized non-surgical management protocols were not used. Non-surgical management included exercise therapy if possible, steroid injections, and/or pharmacological treatments. The non-surgical management modality was left to the discretion of the treating physician and the patient. Baseline demographic variables included age, self-reported back pain (yes/no), radicular leg pain (yes/no), muscle weakness (yes/no), and numbness in the legs (yes/no). In addition, neurological examination was performed (i.e., straight leg raise test, patellar, Achilles, and plantar reflex). Finally, radiographic measures at initial presentation included Cobb angle, location of the apical vertebra, and side of convexity on coronal spine radiographs. Full-spine standing sagittal radiographs were not routinely performed (patient enrollment 1996–2007).

### Data collection

Considering the relative old age of the study population at the current follow-up, a database search was performed in the national database registry for identification of patients that had passed away during follow-up. A Dutch translated and validated version of the Oswestry Disability Index (ODI) 2.0 was sent to eligible patients that had given their written informed consent [18]. In addition, patients were asked if they received (additional) spine surgery in our institution or elsewhere during follow-up. In case of no response, a reminder was sent after 4 weeks.

The ODI is a questionnaire that evaluates the limitations in functional status caused by low back pain. This outcome measure has been shown to be valid, reliable, and responsive in patients with low back pain and is often used in studies in the evaluation of degenerative spinal disorders [19]. The total sum score of the ODI ranges from 0 to 100; the higher the score, the higher the disability. An ODI total score between 0 and 20 reflects minimal disability, 21–40 moderate disability, 41–60 severe disability, 61–80 crippling back pain, and 81–100 bed-bound patients [20]. ODI total score was compared between groups at final follow-up.

Recent study by Van Hooff et al. [21] reported that an absolute ODI score of ≤ 22 reflects an acceptable symptom state and should be considered as a criterion for treatment success in patients who had undergone surgery for degenerative disorders of the lumbar spine. Therefore, the number of patients reporting minimal disability, hence an “acceptable symptom state” (ODI score 0–22) versus severe disability (ODI score 40–100), were stratified according to management.

### Statistical analysis

Data was checked for normal distribution of variables using the Shapiro-Wilk test. Baseline demographic, neurological, and radiographic values were compared between both groups using Student’s *t* test in case of normal distribution of data, whereas a Mann-Whitney *U* test was performed when variables were non-normally distributed. Frequency analysis of categorical variables (ODI score 0–22 versus 40–100) was performed using a Fisher’s Exact test. All statistical tests were performed with SPSS 22.0 IBM. Statistical significance was set at *p* < 0.05.

## Results

### Study population

Thirty (34%) of the 88 patients passed away during follow-up. Six patients’ addresses were untraceable and one patient suffered from a stroke and was therefore not able to answer the questionnaire. Thus, 51 patients were eligible for this long-term follow-up study of which 29 (57%) agreed to participate. Of these 29 patients, 15 (52%) were treated surgically and 14 (48%) were managed non-surgically, with a mean age of 65.2 ± 8.2 (range, min–max 50–81 years) and a median Cobb angle of 19.0 (interquartile range 16.0–25.5°) at initial presentation. The total group consisted out of 22 (76%) females and 7 (24%) males. Mean follow-up was 10.9 ± 1.9 years (range 8–15 years). During follow-up, there was no crossover from non-surgical to surgical group. Baseline demographic, radiological, and clinical data is stratified according to group in Table 1. There was no significant difference between the two groups with regards to age (*p* = 0.25), body mass index (BMI) (*p* = 0.79), Cobb angle (*p* = 0.37), and years of follow-up (*p* = 0.84). Interestingly, the non-surgical group had a larger median coronal Cobb angle than the surgical group (21.0 versus 18.0; *p* = 0.37). With regard to clinical evaluation at initial presentation, all patients (100%) reported back pain and a high comparable number of patients in both groups reported radicular leg pain (79% versus 87%). Neurological symptoms during physical examination (straight leg raise test, patellar, Achilles, and plantar reflex), muscle weakness, and numbness in the legs were found in both groups (Table 1).

**Table 1:** Demographic, radiology, and clinical evaluation at baseline according to surgical and non-surgical management

	All	Non-surgical	Surgical	*p* value
Number of patients	29	14 (48%)	15 (52%)	
Female:Male	22:7	11:3	11:4	
Age at baseline, mean ± SD, (years)	65.2 ± 8.2	67.0 ± 8.8	63.5 ± 7.5	0.253
BMI, mean ± SD, (kg/m^2^)	24.7 ± 3.8	24.3 ± 5.6	24.9 ± 3.2	0.790
Coronal Cobb angle, median [IQR], (°)	19.0 [16.0–25.5]	21.0 [16.0–30.0]	18.0 [16.0–22.0]	0.370
Follow-up time, mean ± SD, (years)	10.9 ± 1.9	10.3 ± 1.5	10.5 ± 1.4	0.836
Apical vertebra, (*n*)				
L1	2	2	0	
L2	12	5	7	
L3	12	6	6	
L4	3	1	2	
Convex side, (*n*)				
Right sided	14	10	4	
Left sided	15	4	11	
Back pain, *n* (%)	29 (100%)	14 (100%)	15 (100%)	
Radicular leg pain, *n* (%)	24 (83%)	11 (79%)	13 (87%)	
Right	11	7	4	
Left	5	1	4	
Left and right	8	3	5	
Not present	5	3	2	
Neurological symptoms^†^, *n* (%)	4 (14%)	1 (7%)	3 (20%)	
Muscle weakness in the legs, *n* (%)	10 (34%)	6 (43%)	4 (27%)	
Right	4	2	2	
Left	3	2	1	
Left and right	3	2	1	
Not present	11	7	4	
Data not available	8	1	7	
Numbness in the legs, *n* (%)	3 (10%)	2 (14%)	1 (7%)	
Yes	3	2	1	
Not present	14	10	4	
Data not available	12	2	10	

Values of age, body mass index (BMI), Cobb angle, and follow-up time are expressed as the mean and standard deviation (normal distribution of data) or as the median and interquartile range [IQR] (non-normal distribution of data). Percentages are calculated from the total number of patients

*P*-value were calculated between non-surgical and surgical group using Student’s *t* test (normal distribution of data) or Mann-Whitney *U* test (non-normal distributed data)

^†^Neurological symptoms evaluated during physical examination by straight leg raise test, patellar, Achilles, and plantar reflex

### Management

Non-surgical management included exercise therapy if possible, steroid injections, and/or pharmacological treatments. All patients treated surgically underwent postero(lateral) lumbar fusion with pedicle screw fixation to restore coronal malalignment and stenosis. Details of surgical treatment are presented in Table 2 and Table 3. Nine (60%) patients underwent long fusion (≥ 4 levels) and six (40%) short fusion (≤ 3 levels). Six (40%) patients underwent additional decompression. Six patients (40%) in the surgical group had one or more revision surgeries during follow-up due to implant failure (3 patients), wound infection (1 patient), or recurrence of symptoms (2 patients). Revision surgery included, but was not limited to, screw replacement, (re-)decompression or extension of the fusion.

**Table 2:** Characteristics of surgical cohort

Parameter	Value
Number of patients	15
Age at index surgery (years)	63.5 ± 7.5
Posterior-only approach	15 (100%)
Posterior instrumentation and fusion	15 (100%)
Number of levels fused, range (average)	2–8 (4.7)
UIV, range	T10-L4
LIV, range	L5-ilium
PLIF	2 (13.3%)
DLIF	2 (13.3%)
Decompression	6 (40%)

Value of age is expressed as the mean and standard deviation

*UIV* upper-most instrumented vertebra, *LIV* lower-most instrumented vertebra, *DLIF* direct lateral interbody fusion, *PLIF* posterior lumbar interbody fusion

**Table 3:** Detailed surgical characteristics at baseline and whether patients underwent revision surgery during follow-up

Baseline						Follow-up
Patient no.	Gender	Age	Cobb (°)	Apical vertebra	Postero(lateral) fusion with pedicle screws	Revision surgery
1	M	71	26°	L2	Th11-S1+ decompression L3-L4	No
2	F	59	37°	L2	Th11-L5	No
3	M	63	18°	L2	L2-L5	Yes
4	F	50	42°	L2	Th10-S1	No
5	F	71	17°	L2	L3-L5 + decompression + DLIF	No
6	F	59	22°	L2	Th12-L5 + PLIF	No
7	F	67	16°	L2	L4-L5+ DLIF	Yes
8	M	66	17°	L3	Th11-S1	Yes
9	F	62	12°	L3	Th12-S1	No
10	M	68	10°	L3	Th12-L5 + decompression L2–3	Yes
11	F	55	22°	L3	Th12-S1	No
12	F	79	22°	L3	Th112-L5 + decompression L4-L5	Yes
13	F	62	19°	L4	L4-L5 + decompression L4-L5	Yes
14	F	66	14°	L4	L3-S1 + decompression L3- S1	No
15	F	54	18°	L3	L5-S1 + PLIF	No

*DLIF* diffuse lateral interbody fusion, *PLIF* posterior lumbar interbody fusion

### Functional outcome

In terms of total score of the ODI, there was no significant difference in the non-surgical versus the surgical group (*p* = 0.649). The non-surgical group had a mean ODI total score of 35.2 ± 26.9 (range 2.0–88.0) and the surgical group a mean ODI total score of 39.4 ± 21.6 (range 4.0–76.0), both groups reflecting an overall moderate disability (ODI range 21–40) [20].

The number of patients reporting minimal disability, hence a “satisfactory symptom state” (ODI total score 0–22) and severe disability (ODI total score 40–100), were stratified according to management in Table 4. More patients in the non-surgical group reported an ODI total score ≤ 22, reflecting minimal disability (43 versus 20%, *p* = 0.245); however, this difference was non-significant. In addition, no significant difference was found in the number of patients reporting severe disability (*p* = 0.715).

**Table 4:** Number of patients reporting minimal disability (ODI score 0–22) versus severe disability (ODI score 40–100) after 10-year follow-up, stratified according to management

ODI total score	Non-surgical (14)	Surgical (15)	*p* value
score 0–22	6 (43%)	3 (20%)	0.245
score 40–100	7 (50%)	6 (40%)	0.715

ODI indicates Oswestry Disability Index; the range is 0–100, with higher numbers reflecting greater disability. ODI total score between 0 and 22 reflects minimal disability hence a “satisfactory symptom state” and an ODI total score 40–100 severe disability. Percentages are calculated from the total number of non-surgical (*n* = 14) and surgical (*n* = 15) patients. *p* values were calculated using Fisher’s Exact Test

## Discussion

In elderly patients, DNDLS significantly affects the overall health-related quality of life and is one of the most prevalent indications for reconstructive spinal surgery [22, 23]. It is important to compare the long-term results of surgical and non-surgical management in order to optimize clinical decision-making. Surgical decompression and spinal correction has shown to improve functional limitations in DNDLS, but long-term follow-up seldom exceeds 2 years. In the current literature, there are no studies that have evaluated both surgical and non-surgical strategies with long-term follow-up. This study provides the first long-term evaluation in functional outcome of non-surgical and surgically treated DNDLS patients. Notable, the purpose of the current analysis was not to suggest that one approach is uniformly superior to the other, but rather to report the functional outcome after long-term follow-up of two management strategies. Despite weaknesses in the methodology (e.g., no baseline sagittal full-spine radiographs), the results of the present study seem to indicate that non-surgical managed patients seem to function reasonably after long periods of time, with surprisingly no crossover to surgery.

The findings of the present study seem somewhat contradictory compared to previous shorter term follow-up studies which have suggested that surgical management is superior to non-surgical management in terms of pain relief and improved function [11, 14–16]. In a retrospective study, Dickson et al. [24] compared 81 patients who underwent surgical treatment versus 30 patients that underwent non-surgical management. Patients in the surgical group demonstrated significantly more improvement of pain, fatigue, and disability over 5 years of follow-up, compared to the non-surgical managed group. However, the study by Dickson and colleagues had several limitations, including the use of the traditional Harrington distraction rod instrumentation and non-validated outcome measures. Smith et al. [16] offered perhaps the most complete assessment of the benefit of surgical treatment as compared to non-surgical management in adult spinal deformity. In a multicentre prospective analysis, surgical and non-surgical treated patients were matched based upon similar radiographic and clinical features. The surgical treated group demonstrated significant improvements in Scoliosis Research Society-22 questionnaire (SRS-22), ODI, and Numeric Rating Scale (NRS) back and leg pain scores at 2 years follow-up, whereas the non-surgical cohort maintained the baseline level of pain and disability scores. However, whether 2-year outcome evaluation is adequate in adult spinal deformities is debatable [25]. Bridwell et al. [26] evaluated changes in radiographic and clinical outcome of primary adult scoliosis surgery after 2, 3, and 5 years’ follow-up. In a multicentre prospective analysis, Bridwell et al. [26] demonstrated that patients who experienced new complications (i.e., implant failure, infection, and proximal junctional failure) that were identified at final follow-up demonstrated significantly worse ODI and SRS total scores. For this reason, it is important to evaluate the durability of surgical and non-surgical management over longer term follow-up and to define which patients may benefit most from which treatment regimen.

In the present study, we sought to assess one important patient-reported outcome measure to provide insight in the long-term results of both management regimens. We chose to focus on the ODI as it is considered the gold standard to assess functional limitations caused by low back pain, a characterizing feature of DNDLS [20]. At initial presentation, all patients reported some degree of back pain; radicular leg pain was reported by 79% in the non-surgical group and 87% in the surgical group. In addition, a moderate number of patients demonstrated pre-treatment weakness in the legs in the non-surgical group and in the surgical group (43 and 27%, respectively) (Table 1). After a mean follow-up of 10 years, no significant difference was found in ODI scores between surgical and non-surgical management in DNDLS. Moreover, a higher proportion of patients report an acceptable symptom state (ODI total score 0–22) in the non-surgical group compared to the surgical group (43 versus 20%); however, this difference was non-significant (*p* = 0.245) (Table 4). Even though the studied patient cohort (e.g., relatively small Cobb angle, a high percentage of reported back and leg pain, and pre-treatment neural comprise) in the present study is relatively comparable to previous studies demonstrating that surgical is superior to non-surgical management (Table 1), the results of the present study demonstrate that certain patients can benefit from non-surgical management for long periods of time (Table 4). Further study is warranted to determine which patients benefit most from which treatment regimen.

The results of the present study should be interpreted with some caution. The objective of the present study was to evaluate long-term functional outcome of both strategies, and thus, it was not designed to assess the gain in functional outcome over time, since it does not include ODI assessment at initial presentation. Despite great efforts to obtain the follow-up measures in both groups, many patients (34%) had passed away during follow-up or did not want to participate (25%). The difficulty of including non-surgical managed elderly patients in a follow-up study has previously been reported. Previous studies achieved a 2-year follow-up rate of 45–55% when including non-operative patients [15, 27]. The present study achieved a total follow-up rate of 57% (excluding deceased patients) after a mean follow-up exceeding 10 years. The major limitations of the present study are related to its potential for selection bias (despite the fact that we were not able to identify any significant differences between the groups), the relatively small follow-up rate, and the absence of data related to other forms of non-surgical management (protocols were not used) between baseline and follow-up that patients may have tried. Finally, the surgical correction of adult spinal deformity, including restoration of spinopelvic alignment and sagittal balance, is now better defined [28]. However, the present study could not take into account sagittal spinopelvic alignment due to the historic context of the cohort (patient enrolment 1996–2007) and the absence of lateral full-spine standing radiographs at initial presentation. For this reason, we cannot make direct conclusions regarding the long-term surgical outcome of restoring sagittal spinopelvic alignment. Whether restoring sagittal alignment will prove significant beneficial on the longer term will require further study and careful follow-up.

## Conclusions

This is the first study that describes the long-term outcome of non-surgical and surgical management in patients with symptomatic DNDLS. For these relatively comparable cohorts of patients from a single institution and during the same time frame, we could not demonstrate a significant difference in ODI total score between surgical and non-surgical management after long-term follow-up exceeding 10 years. Despite methodological weaknesses, this study provides the first ever long-term evaluation in patient reported outcome of non-surgical and surgical treated symptomatic DNDLS patients. The results indicate that certain patients can benefit from non-surgical management after long periods of time. Further study is warranted to determine which patients benefit most from which treatment regimen.

## Abbreviations

BMI: Body mass index
DNDLS: De novo degenerative lumbar scoliosis
NRS: Numeric Rating Scale
ODI: Oswestry Disability Index
SRS-22: Scoliosis Research Society-22 Questionnaire

## References

[1] Aebi M (2005). The adult scoliosis. Eur Spine J.

[2] Smith JS, K-M F, Urban P, Shaffrey CI (2008). Neurological symptoms and deficits in adults with scoliosis who present to a surgical clinic: incidence and association with the choice of operative versus nonoperative management—clinical article. J Neurosurg Spine.

[3] Bess S, Boachie-Adjei O, Burton D (2009). Pain and disability determine treatment modality for older patients with adult scoliosis, while deformity guides treatment for younger patients. Spine (Phila Pa 1976).

[4] Longo DL, Ropper AH, Zafonte RD (2015). Sciatica. N Engl J Med.

[5] Perennou D, Marcelli C, Herisson C, Simon L (1994). Adult lumbar scoliosis. Epidemiologic aspects in a low-back pain population. Spine (Phila Pa 1976).

[6] Schwab F, Dubey A, Gamez L (2005). Adult scoliosis: prevalence, SF-36, and nutritional parameters in an elderly volunteer population. Spine (Phila Pa 1976).

[7] United States Census Bureau 2014. http://www.census.gov/topics/population/age-and-sex.html. Accessed 31 Dec 2016.

[8] The Nationwide Inpatient Sample, US (2014) http://hcup-us.ahrq.gov/nisoverview.jsp Accessed 11 Sept 2016.

[9] Glassman SD, Berven S, Kostuik J (2006). Nonsurgical resource utilization in adult spinal deformity. Spine (Phila Pa 1976).

[10] Ames CP, Scheer JK, Lafage V (2016). Adult spinal deformity: epidemiology, health impact, evaluation, and management. Spine Deform.

[11] Smith JS, Shaffrey CI, Berven S (2009). Operative versus nonoperative treatment of leg pain in adults with scoliosis: a retrospective review of a prospective multicenter database with two-year follow-up. Spine (Phila Pa 1976).

[12] Glassman SD, Schwab FJ, Bridwell KH (2007). The selection of operative versus nonoperative treatment in patients with adult scoliosis. Spine (Phila Pa 1976).

[13] Daubs MD, Lenke LG, Cheh G (2007). Adult spinal deformity surgery: complications and outcomes in patients over age 60. Spine (Phila Pa 1976).

[14] Smith JS, Shaffrey CI, Berven S (2009). Improvement of back pain with operative and nonoperative treatment in adults with scoliosis. Neurosurgery.

[15] Bridwell KH, Glassman S, Horton W (2009). Does treatment (nonoperative and operative) improve the two-year quality of life in patients with adult symptomatic lumbar scoliosis: a prospective multicenter evidence-based medicine study. Spine (Phila Pa 1976).

[16] Smith JS, Lafage V, Shaffrey CI (2016). Outcomes of operative and nonoperative treatment for adult spinal deformity: a prospective, multicenter, propensity-matched cohort assessment with minimum 2-year follow-up. Neurosurgery.

[17] de Vries B, Mullender MG, Pluymakers WJ (2010). Spinal decompensation in degenerative lumbar scoliosis. Eur Spine J.

[18] van Hooff ML, Spruit M, Fairbank JCT (2015). The Oswestry Disability Index (version 2.1a): validation of a Dutch language version. Spine (Phila Pa 1976).

[19] Fairbank JC, Couper J, Davies JB, O’Brien JP (1980). The Oswestry low back pain disability questionnaire. Physiotherapy.

[20] Fairbank JC, Pynsent PB (2000). The Oswestry Disability Index. Spine (Phila Pa 1976).

[21] van Hooff ML, Mannion AF, Staub LP (2016). Determination of the Oswestry Disability Index score equivalent to a “satisfactory symptom state” in patients undergoing surgery for degenerative disorders of the lumbar spine-a Spine Tango registry-based study. Spine J.

[22] Healthcare Costs, Utilization Project. U.S. Department of Health and Human Services; 2013. Available at: https://www.hcup-us.ahrq.gov/; Accessed 28 July 2016.

[23] Terran J, McHugh B, Fischer C (2014). Surgical treatment for adult spinal deformity: projected cost effectiveness at 5-year follow-up. Ochsner J.

[24] Dickson JH, Mirkovic S, Noble PC (1995). Results of operative treatment of idiopathic scoliosis in adults. J Bone Joint Surg Am.

[25] Glassman SD, Schwab F, Bridwell KH (2009). Do 1-year outcomes predict 2-year outcomes for adult deformity surgery?. Spine J.

[26] Bridwell KH, Baldus C, Berven S (2010). Changes in radiographic and clinical outcomes with primary treatment adult spinal deformity surgeries from two years to three- to five-years follow-up. Spine (Phila Pa 1976).

[27] Smith JS, Shaffrey CI, Berven S (2009). Improvement of back pain with operative and nonoperative treatment in adults with scoliosis. Neurosurgery.

[28] Schwab FJ, Blondel B, Bess S (2013). Radiographical spinopelvic parameters and disability in the setting of adult spinal deformity: a prospective multicenter analysis. Spine (Phila Pa 1976).

